# Eczema

**Published:** 1880-08

**Authors:** 


ECZEMA.

Dr. Lambart, in the London *Lancet*, states that within the last twelve months two cases of eczema have come under his observation, and both have yielded to the following treatment: First, the affected part was painted with pure carbolic acid; as soon as this became dry, collodion was similarly applied. This was repeated every fifth day. At the same time solution of arsenic was administered three times daily.

